# Systematic review and meta-analysis of risk factor for postoperative delirium following spinal surgery

**DOI:** 10.1186/s13018-020-02035-4

**Published:** 2020-11-05

**Authors:** Hao Jie Zhang, Xue Hai Ma, Jin Biao Ye, Cong Zhi Liu, Zhi Yang Zhou

**Affiliations:** Department of Orthopedics, Huai An Hospital of Huai An City, No. 161, Zhenhuailou East Road, Huai’an District, Huai’an City, Jiangsu Province, 223200 China

**Keywords:** Delirium, Spinal surgery, Risk factor, Meta-analysis

## Abstract

**Background:**

Postoperative delirium is a common psychiatric disorder among patients who undergo spinal surgery. The purpose of current meta-analysis was to assess the potential risk factors related to delirium in spinal surgery.

**Methods:**

We searched the following databases: PubMed, EMBASE, the Cochrane Library, and Web of Science, from inception to July 2020. Two reviewers independently assessed the quality of the included studies using the previously described Newcastle-Ottawa Scale (NOS). We included spinal surgery patients who suffered with delirium or not. Stata 12.0 was used for meta-analysis.

**Results:**

Thirteen trial studies that met our inclusion criteria were incorporated into the meta-analysis. Postoperative delirium was associated with an increase of the duration of hospital stay (*P* = 0.044) and increased perioperative readmission rate (*P* = 0.013) and economic costs (*P* = 0.002). This meta-analysis demonstrates that there were twenty-two risk factors: general characteristic: old age, female patients, history of surgery, diabetes mellitus, hypertension; preoperative data: low hematocrit, low hemoglobin, low albumin, low sodium, depression; operative data: operating time, total blood loss; postoperative data: low sodium, low hemoglobin, low hematocrit, low albumin, fever, low potassium, blood sugar, and visual analog scale (VAS).

**Conclusions:**

Delirium not only prolongs the length of hospital stay, but also increases readmission rate and the economic costs. Several risk factors including old age, female patients, history of surgery, diabetes mellitus, low hematocrit, low hemoglobin, low albumin, low sodium, depression; operative data: operating time, total blood loss, low sodium, low hemoglobin, low hematocrit, low albumin, fever, low potassium, blood sugar, and VAS were significant predictors for postoperative delirium after spinal surgery.

## Background

Delirium is defined as an acute disorder of attention and cognition and is associated with underlying physiological disorders [1]. Postoperative delirium after spinal surgery was a common complication in older patients. The incidence of postoperative delirium after spinal surgery has been widely reported in the literature and ranges from 18.4 to 40.5% [2]. Patients with delirious state were associated with a prolonged hospital stay, increased the economic costs, and impaired individual’s function and quality of life [3]. Postoperative delirium occurs in many surgeries, including major vascular surgery, hip surgery, and spinal surgery [4, 5]. However, the mechanism of delirium was unclear. Thus, understanding the risk factor for delirium in spinal patients was important.

A meta-analysis review by Shi et al. [6] evaluated the risk factor of delirium after spinal surgery. However, it contained some methodological shortcomings; all of the risk factors were not evaluated and with high heterogeneity. Not only did these studies have these limitations, but also they did not individually separate preoperative hemoglobin level and postoperative hemoglobin level. Considering all these issues and new evidence emerging, it is impossible to give clear advice on the risk factor of the delirium after spinal surgery. Thus, we undertook a further meta-analysis to identify the risk factors for delirium in spinal surgery patients.

## Methods

This study was designed and reported according to the Preferred Reporting Items for Systematic Reviews and Meta-Analyses (PRISMA) statement [7].

### Literature search

We searched the following electronic databases: PubMed, EMBASE, the Cochrane Library, and Web of Science from inception to July 2020. The search terms were [delirium OR confusion OR transient mental disorder OR dementia OR cognitive disorders] AND [spine OR spinal OR lumbar infusion] AND [surgery OR operation]. We also searched the references of the included studies and recent reviews or meta-analysis.

### Study selection

Inclusion criteria were as follows: (i) patients who had undergone spinal surgery; (ii) the studies’ design was observational or cohort study; (iii) delirium and controls diagnosed by delirium assessment tool; (iv) studies reporting adequate data for pooling for the analysis; (v) studies published in Chinese or English. The exclusion criteria were as follows: (i) review articles, letter, or comments; (ii) studies without available data for statistics; (iii) there were no diagnostic criteria for delirium after spinal surgery.

### Data extraction

All the data were extracted by two authors from all eligible studies (Hao-Jie Zhang and Xue-Hai Ma). The following variables were extracted from each study: first author’s name, publication year, country, study design, study setting, sample size, age, sex ratio, criteria for delirium, screening frequency, study quality score. Any disagreement was resolved by discussion or consulted from a senior reviewer to reach a consensus.

### Quality assessment

Two reviewers (Jin-Biao Ye and Cong-Zhi Liu) independently assessed the quality of the included studies using the previously described Newcastle-Ottawa Scale (NOS) [8]. A total of three items were included: (1) patient selection, (2) comparability of the two study arms, and (3) assessment of the outcomes. Studies were classified according to quality: high quality (7–9), moderate quality (5–6), poor quality (0–4).

### Data synthesis and analysis

All statistical analyses were conducted in Stata 12.0 (Stata Corp., College Station, TX). We estimated the pooled risk ratios (RRs) for the binary variable and weighted mean differences (WMD) for the continuous data. We used random effects model or fixed effects model according to the heterogeneity between the studies. We assessed statistical heterogeneity using the *I* square (*I*^2^) values (*I*^2^ > 50% was considered to imply statistical heterogeneity).

## Results

**Fig. 1:**
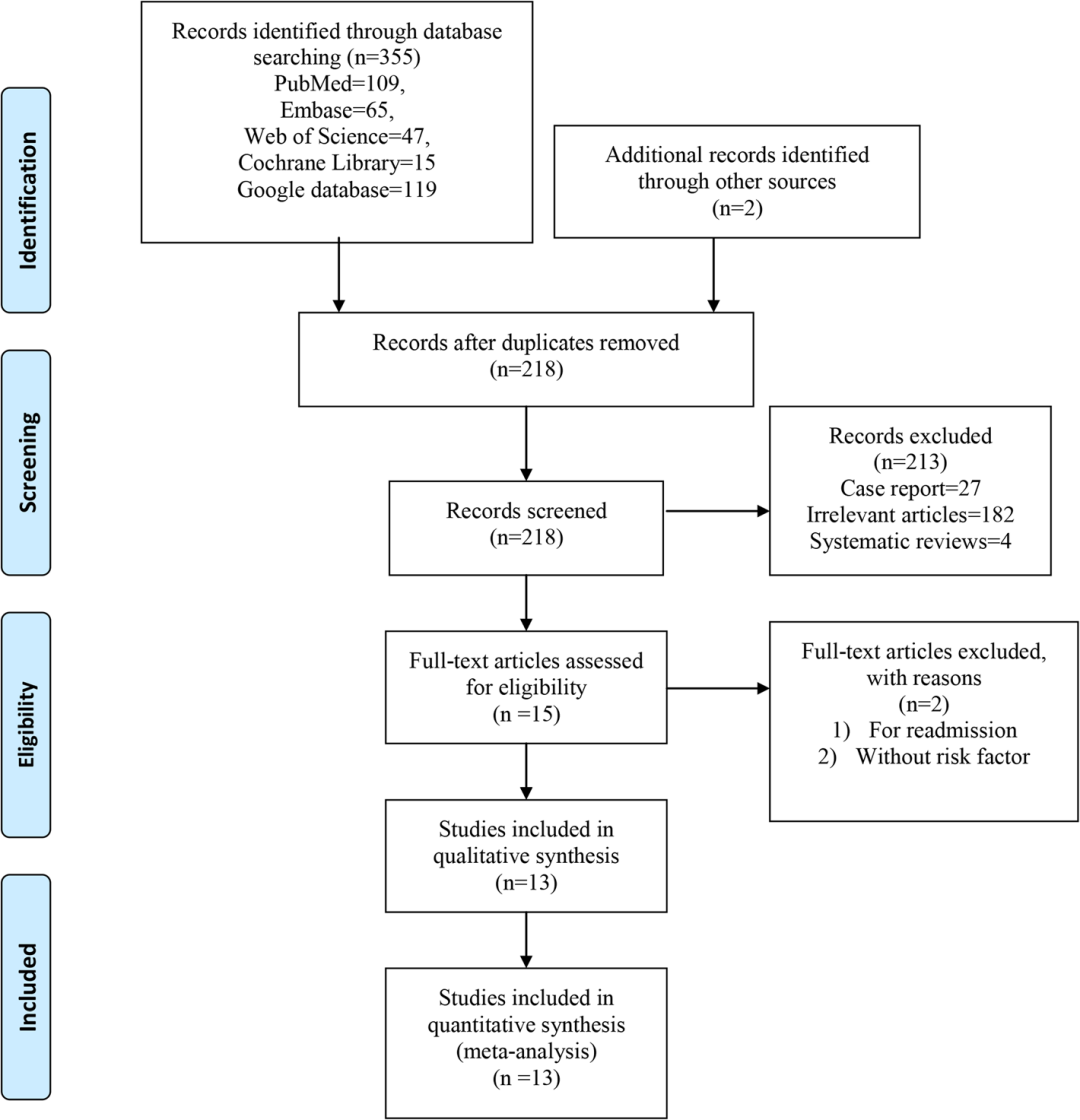
The flow diagram of the study identification and inclusion and exclusion process

First, we initially yielded 355 relevant studies from electronic databases and 2 additional records through other sources, of which 137 publications were excluded because they were duplications. After reading the title and abstract of these 218 papers, 203 papers were excluded as they did not fulfill the inclusion criteria. Then, 2 studies were excluded after reading the full-text; one study was not interested in delirium but for readmission; another study did not include risk factor of delirium. Ultimately, thirteen trials [9–21] met the inclusion criteria. The flow diagram of study selection is shown in Fig. 1.

### Study characteristics and quality assessment

General characteristics of the included studies are presented in Table 1. The publication year of the 24 studies ranged from 2004 to 2018. Four studies were published in the USA, three studies originated from China, three originated from Japan, and the rest were from Korea. Study quality score ranged from 6 to 9.

**Table 1:** The general characteristic of the included studies

Reference	Country	Study design	Study setting	Sample size	Age (year)	Sex ratio (M:F)	Criteria for delirium	Screening frequency (h)	Study quality score
Adogwa et al. [9]	USA	RCS	Correction of adult degenerative scoliosis	82	71.8	NS	CAM	n.s	7
Brown et al. [10]	USA	Cohort	Lumbar spine surgery, posterior cervical spine surgery, or anterior cervical spine	89	74	47/42	CAM	24 h	9
Elsamadicy et al. [11]	USA		Elective spine surgery				NS		7
Fineberg et al. [12]	USA	RCS	Lumbar spine	578,457			ICD-9-CM	NS	6
Gao et al. [13]	China	RCS	Cervical, thoracic, lumbar, and sacral spine	549	48.2		DOS, DSM-IV	24 h	6
Jiang et al. [14]	China	RCS	Fusion, decompressive laminectomy and discectomy of the lumbar spine, and anterior cervical discectomy and fusion, laminoplasty, and foraminotomy of the cervical spine	451	65.1	226/225	NS	NS	6
Kawaguchi et al. [15]	Japan	RCS	Cervical spine, cervico-thoracic spine in the thoracic spine, in the thoraco-lumbar spine, in the lumbar spine, and cervical and lumbar spine	341	59.2	186/155	CAM	NS	6
Kobayashi et al. [16]	Japan	RCS	Cervical, thoracic, and lumbar spine	262	82.7	122/140	CONFUCIUS stepped wedge protocol	NS	8
Lee et al. [17]	Korea	RCS	Anterior fusion and posterolateral fusion	81	73.5	28/53	DSM-IV, CAM	NS	8
Seo et al. [18]	Korea	RCS	Operation, decompressive laminectomy and discectomy of the lumbar spine, anterior cervical discectomy and fusion, laminoplasty and foraminotomy of the cervical spine	70	70.1	32/38	DSM-5	NS	9
Ushida et al. [19]	Japan	RCS	Cervical myelopathy	81	NS	41/41	NS	NS	8
Li et al. [20]	China	RCS	Spinal surgery	116	75.3	62/54	CAM-ICU	24 h	6
Adogwa et al. [21]	USA	RCS	Spinal deformity surgery	82	74.7	64/18	CAM-ICU	NS	8

*RCS* retrospective controlled study, *CAM-ICU* Confusion Assessment Method for the Intensive Care Unit, *CAM* Confusion Assessment Method, *DSM-5* Diagnostic and Statistical Manual of Mental Disorders-5

### Results of meta-analysis

**Fig. 2:**
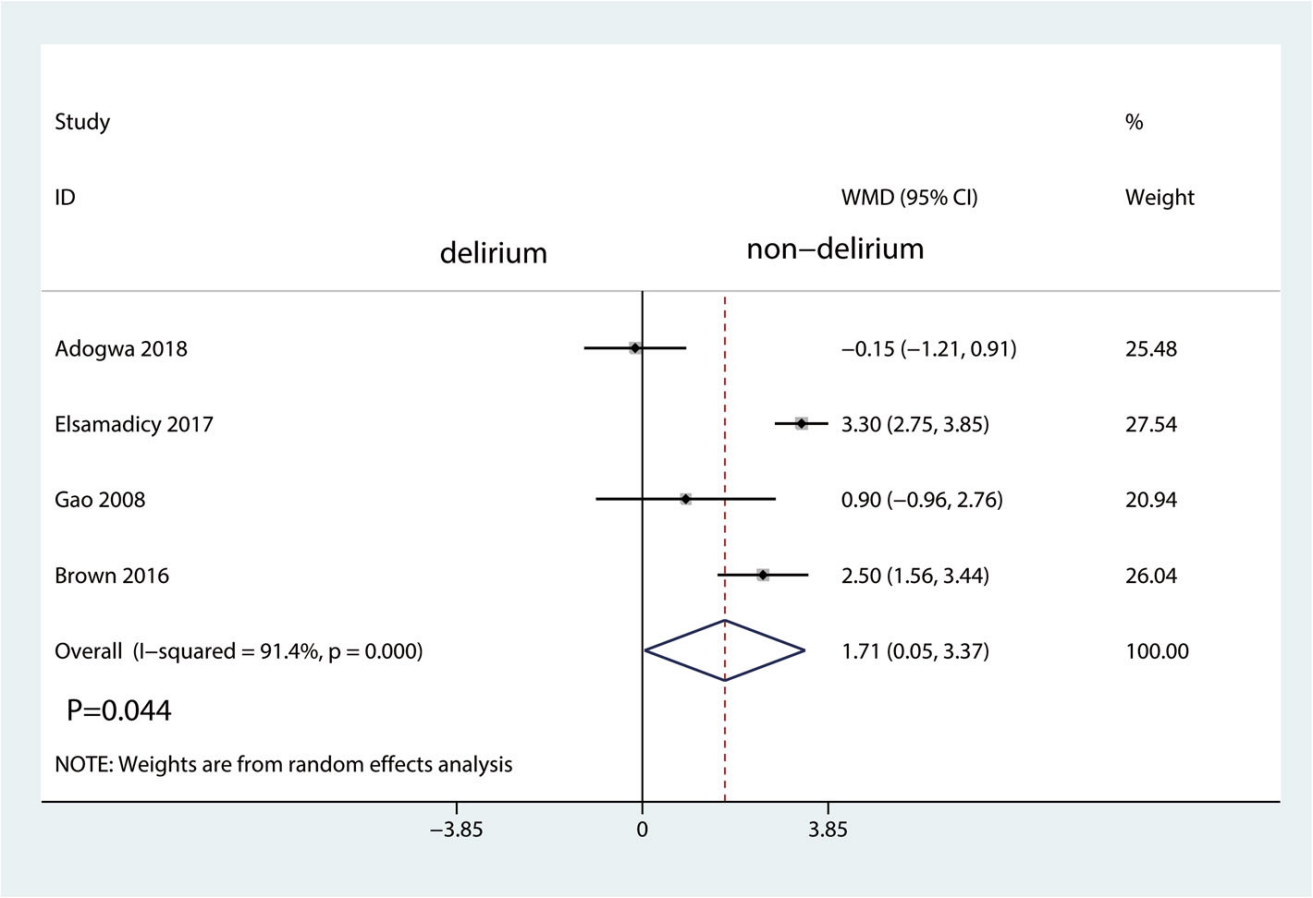
The forest plot for duration of hospital stay in the delirium group versus the non-delirium group

**Fig. 3:**
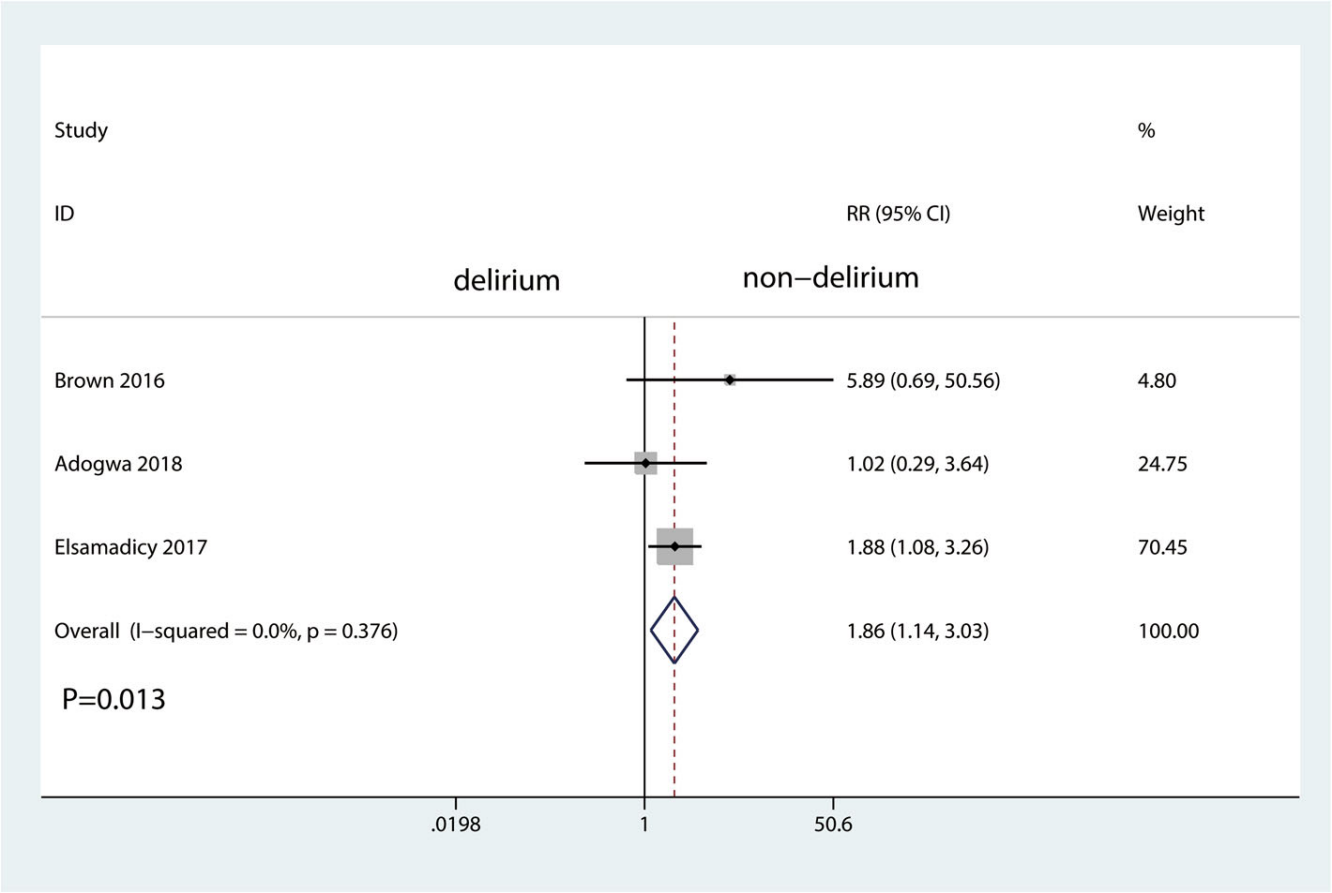
The forest plot for readmission rate in the delirium group versus the non-delirium group

**Fig. 4:**
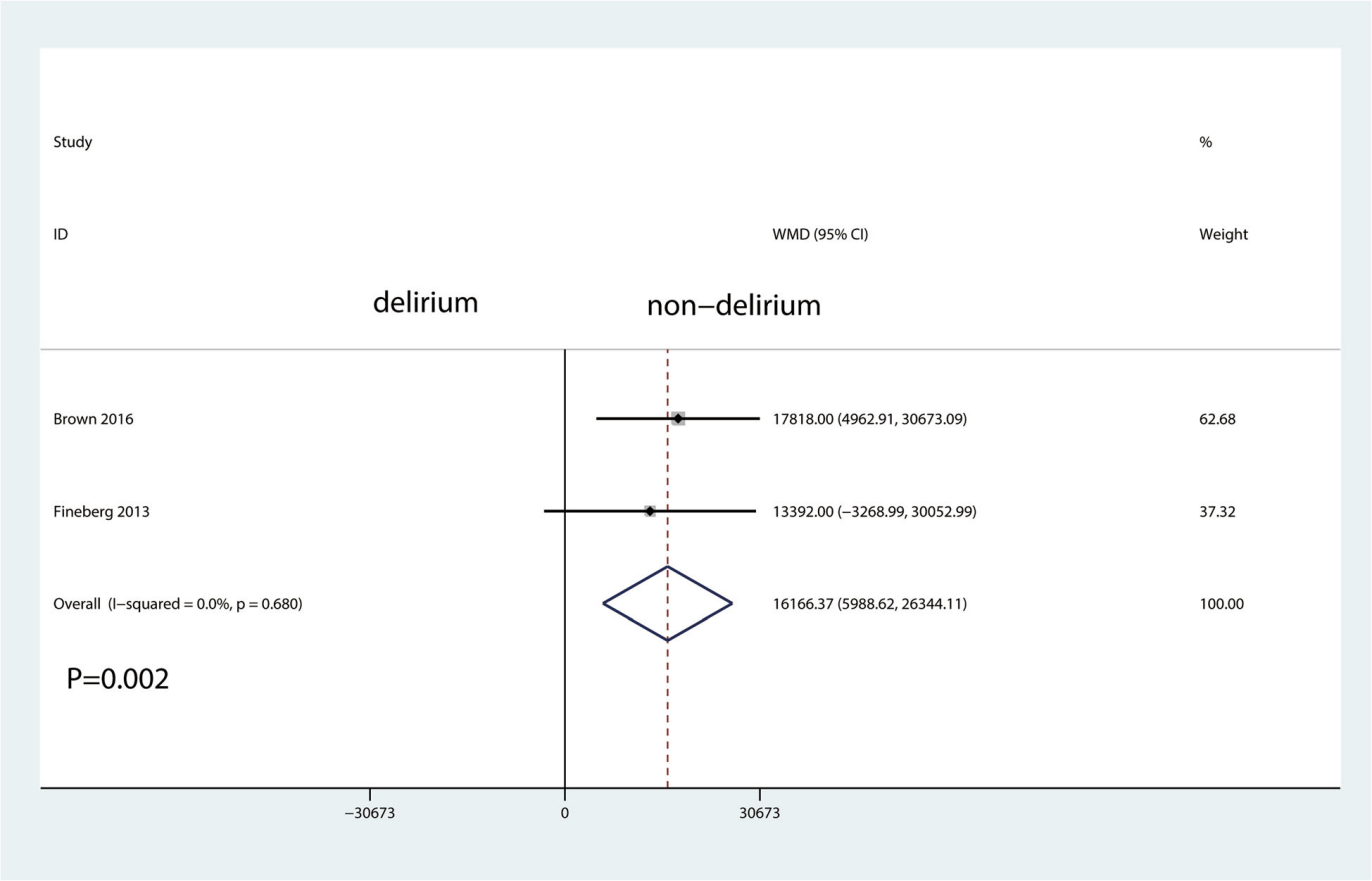
The forest plot for economic costs in the delirium group versus the non-delirium group

There was also a statistically significant association of delirium with increased duration of hospital stay (MD = 1.71 (95% CI 0.05 to 3.37) days; *P* = 0.044; Fig. 2) and increased perioperative readmission rate (RR 1.86, 95% CI 1.14 to 3.03, *P* = 0.013, Fig. 3) and economic costs (MD = 16166.37 (95% CI 5988.62 to 26,344.11) *P* = 0.002, Fig. 4).

### Risk factors

Patients who developed delirium were significantly older (age > 65, RR = 6.13 (95% CI 5.75, 6.54), *P* = 0.000; age > 70, RR = 18.23 (95% CI 3.13, 107.34), *P* = 0.001, mean age, WMD = 1.34 (95% CI 0.34, 2.33), *P* = 0.009, Table 2). Sex was also associated with delirium in univariable analysis or meta-analysis (female patients, RR = 1.21 (95% CI 1.15, 1.28, Table 2)).

**Table 2:** Meta-analysis of risk factors for postoperative delirium in older spinal surgical patients

Risk factor	Studies	Statistically methods	RR or WMD with 95%Cis	I^2^(%)	*P* value
**Preoperative**					
Age > 65 years	2	IV, fixed	6.13 (5.75, 6.54)	0.0	0.000
Age > 70 years	3	IV, random	18.23 (3.13, 107.34)	48.4	0.001
Sex (% female)	8	IV, fixed	1.21 (1.15, 1.28)	0.001	0.000
Mean age	6	IV, fixed	1.34 (0.34, 2.33)	6.0	0.009
Number of medication	3	IV, random	0.92 (− 0.05, 1.88)	77.5	0.063
Surgical history	6	IV, fixed	2.22 (1.47, 3.34)	0.0	0.000
Diabetes mellitus	7	IV, random	2.80 (1.15, 6.79)	70.6	0.023
Cerebral vascular diseases	3	IV, fixed	3.18 (1.27, 7.79)	15.7	0.014
Low hematocrit (%)	3	IV, fixed	− 1.66 (− 2.94, − 0.38)	0.0	0.011
Low hemoglobin (g/L)	4	IV, fixed	− 0.41 (− 0.75, − 0.07)	0.0	0.017
Low albumin (g/dL)	2	IV, fixed	− 0.30 (− 0.50, − 0.11)	18.7	0.003
Low sodium	4	IV, random	− 1.81 (− 3.33, − 0.30)	77.8	0.019
Low potassium	2	IV, random	0.00 (− 0.28, 0.28)	45.4	0.996
Blood sugar	2	IV, random	0.13 (− 0.52, 0.78)	33.4	0.698
Hypertension	7	IV, random	2.01 (1.04, 3.88)	58.3	0.038
Depression	2	IV, fixed	2.02 (1.00, 4.06)	0.0	0.049
**Intraoperative**					
Operating time	9	IV, random	29.42 (1.12, 57.72)	87.9	0.042
Total blood loss	8	IV, fixed	116.77 (93.66, 139.88)	14.9	0.000
Fusion level	2	IV, random	0.35 (− 0.72, 1.42)	52.3	0.524
Intravenous fluids	4	IV, random	231.24 (− 137.99, 600.47)	68.9	0.220
**Postoperative data**					
Low sodium (mEq/L)	4	IV, random	− 3.05 (− 5.47, − 0.63)	89.7	0.014
Low hemoglobin (g/L)	6	IV, random	− 0.76 (− 1.24, − 0.29)	82.5	0.002
Low hematocrit (%)	3	IV, fixed	− 2.53 (− 3.59, − 1.47)	0.0	0.000
Low albumin (g/dL)	2	IV, fixed	− 0.10 (− 0.27, 0.07)	0.0	0.245
Fever	2	IV, fixed	4.52 (2.94, 6.95)	0.0	0.000
Low potassium	4	IV, random	− 3.05 (− 5.47, − 0.63)	89.7	0.014
Blood sugar	1	IV, fixed	1.00 (1.00, 1.96)	0.0	0.041
VAS	2	IV, random	0.98 (0.11, 1.85)	55.4	0.027

Surgical history was also a risk factor of delirium (RR = 2.22; 95% CI 1.47, 3.34; Table 2). Diabetes mellitus and hypertension were also risk factors of delirium (RR = 2.80, 95% CI 1.15, 6.79), *P* = 0.023; RR = 2.01, 95% CI 1.04, 3.88, *P* = 0.038, Table 2). Low hematocrit, low hemoglobin, low albumin, and low sodium were the risk factors of delirium (*P* = 0.011, *P* = 0.017, *P* = 0.003, and *P* = 0.019, Table 2). We also found that depression was the risk factor of delirium of patients undergoing spinal surgery (RR = 2.02; 95% CI 1.00, 4.06; *P* = 0.049; Table 2).

### Intraoperative data

For intraoperative data, we revealed that operating time (WMD = 29.42; 95% CI 1.12, 57.72; *P* = 0.042, Table 2) and total blood loss (WMD = 116.77; 95% CI 93.66, 139.88; *P* = 0.000; Table 2) were risk factor of delirium.

### Postoperative data

Based on the combined RRs or WMDs, we identified the following risk factors: low sodium (WMD = − 3.05; 95% CI − 5.47, − 0.63; *P* = 0.014; Table 2), low hemoglobin (WMD = − 0.76; 95% CI − 1.24, − 0.29; *P* = 0.002; Table 2), low hematocrit (WMD = − 2.53; 95% CI − 3.59, − 1.47; *P* = 0.000; Table 2), fever (WMD = 4.52; 95% CI 2.94, 6.95; *P* = 0.000; Table 2), low potassium (WMD = − 3.05; 95% CI − 5.47, − 0.63; *P* = 0.014; Table 2), blood sugar (WMD = 1.00; 95% CI 1.00, 1.96; *P* = 0.041; Table 2), and VAS (WMD = 0.98; 95% CI 0.11, 1.85, *P* = 0.027; Table 2).

## Discussion

Thirteen studies met the inclusion criteria and investigated risk factors for postoperative delirium in older people undergoing spinal surgery. Results in this meta-analysis suggested the overall prevalence of postoperative delirium was 11.5%. The prevalence of delirium ranged from 3.8 to 40.4% in the included studies.

Postoperative delirium was associated with increased duration of hospital stay, readmission rate, and the economic costs. Scholz et al. [22] found that patients with postoperative delirium had a significantly increased duration of hospital stay compared with those without delirium.

A better understanding of risk factors for delirium may allow stratification of patients before surgery, enabling targeting of interventions and healthcare resources, for example the Comprehensive Geriatric Assessment, a proven multidisciplinary intervention that can improve outcomes in patients with postoperative delirium [23].

This meta-analysis demonstrates that twenty-two risk factors: general characteristic: old age, female patients, history of surgery, diabetes mellitus, hypertension; preoperative data: low hematocrit, low hemoglobin, low albumin, low sodium, depression; operative data: operating time, total blood loss; postoperative data: low sodium, low hemoglobin, low hematocrit, low albumin, fever, low potassium, blood sugar, and VAS.

One of the most important risk factors was advanced age, especially patient over 70 years old are more likely to experience postoperative delirium than those relatively young. Shi et al. [6] revealed that age > 65 years was the risk factor for delirium. We further analyze that the age > 70 was more likely to subject to delirium. Zhu et al. [24] conducted a meta-analysis and found that old age, age > 70 years, was a risk factor of the major head neck cancer surgery. This may be due to the fact that elderly patients were more likely influenced by age-related physical and psychical changes, such as poor organ compensative capacity, reduced body adaptability, and declined adjustment ability. Watt et al. [25] found that incidence of postoperative delirium was 18.4% in elective surgery. What’s more, they revealed that psychotropic medication use and smoking status were two risk factors for delirium in elective surgery patients.

Patients who developed postoperative delirium were more often females. Our results were opposite of previous meta-analysis. Zhu et al. [24] deemed that women could deal with postoperative psychological stress better than male and thus was associated with less delirium. Shi et al. [6] identified female patients were associated with more delirium than male patients. Scholz et al. [22] found that sex was not associated with delirium in univariable analysis or meta-analysis. Current meta-analysis also found that hypotension was the risk factor of delirium after spinal surgery. Scholz et al. [22] found that intraoperative hypotension and perioperative blood transfusion were the risk factors of delirium.

Low hemoglobin and low hematocrit may reduce oxygen supply to the brain and thus causing delirium. Marcantonio et al. [26] also suggest that the postoperative levels of hematocrit should be kept at 30% or higher through appropriate transfusion in order to prevent postoperative delirium. Meanwhile, the low preoperative levels of albumin seem to indicate poor nutritional condition before surgery and after surgery, like the case with gastric ulcer as comorbidity [17].

There were several limitations in this meta-analysis: (1) heterogeneity bias within the selected studies. In particular, the review was susceptible to heterogeneity owing to the inclusion of different spinal surgery (cervical spondylosis, spinal scoliosis, and lumbar degenerative disease). (2) Another limitation was that only articles published in English and Chinese were included, yet most of the studies were undertaken in non-English-speaking countries. We also did not include unpublished papers and thus may have selection bias. (3) All studies assessed delirium from 3 to 7 days after surgery. Differences in duration of follow-up may have introduced bias, but the majority used 5 days and consistently demonstrated the highest rate of delirium in the first 3 days.

## Conclusion

Delirium not only prolongs the length of hospital stay, but also increases readmission rate and the economic costs. Several risk factors including old age, female patients, history of surgery, diabetes mellitus, low hematocrit, low hemoglobin, low albumin, low sodium, depression; operative data: operating time, total blood loss, low sodium, low hemoglobin, low hematocrit, low albumin, fever, low potassium, blood sugar, and VAS were significant predictors for postoperative delirium after spinal surgery. Early identification of these factors is warranted for improving patient outcomes.

## Abbreviations

CNKI: China National Knowledge Infrastructure
RRs: Risk ratios
CIs: Confidence intervals
WMD: Weighted mean difference
PRISMA: Preferred Reporting Items for Systematic Reviews and Meta-Analyses
NOS: Newcastle-Ottawa Scale
VAS: Visual analog scale
